# Atomized Inhalations in Diseases of the Air Passages and Lungs

**Published:** 1867-09

**Authors:** W. M. Cornell

**Affiliations:** Boston


### Correspondence.

### Atomized Inhalations in Diseases of the Air Passages and Lungs.

BY W. M. CORNELL, M. D., LL.D., OF BOSTON.

To Dr. J. F. Miner, Editor of the Buffalo Medical and Surgical Journal:

Dear Sir:—I have read your valuable Journal for several years with pleasure and profit, and as I always wish to do something for something, I send you the following, which you may give to your readers if you deem it worthy:

Some family characteristic early called my special attention to this class of diseases, belonging, as I did, to a consumptive family.

In 1850, I wrote some twenty or more pages upon this subject, or rather upon the "Inhalation of Vapors and Powders" in these diseases, then published in the *Boston Medical and Surgical Journal.* In those I gave the experience of the profession in inhalation, down to that period, with my own, in experiments with various remedies, among which the following notices may be found in the edition of the U. S. Dispensatory for 1850, by Wood & Bache, under the article, "Argentum—Nitrate of silver in impalpable powder, mixed with an equal weight of lycopodium, and used by inhalation, has been found beneficial in ulcerated sore throat, laryngetis, bronchitis, and incipient phthisis, by Dr. W. M. Cornell, of Boston." In the edition of the same work, of 1865, page 1014, the same notice is continued, with this addition: "The salt, used in this way, has since been successfully employed in the treatment of chronic laryngitis, by M. Trousseau, of Paris, and others." In the same manner I then used alum, and various other medicinal articles, with considerable success.

I now refer to these facts merely to remind your readers that I have long paid special attention to this mode of treatment in this class of diseases. Still, I wish it to be understood that I have never employed *inhalation* to the exclusion of general treatment, when it was indicated. However beneficial one kind of treatment may be in some cases, I do not believe in making it a *hobby*, or relying upon it solely in all cases. Recently, you are aware, an improvement has been made in inhaling *atomized* fluids, and the special object of this paper is to state the experience which I have had with this mode of inhalation. The remedies used with the atomized are the same that I used in powders and vapors; but the atomizer, I acknowledge to be an improvement upon my former plan, which had been adopted by Trousseau and others. I did not invent the atomizer, but I readily adopted it as an improvement upon what I did invent or try. I have had under treatment the following cases to report:

1.—Myself. It is now thirty years since I left the pastoral charge on account of a chronic debility of the vocal chords, which caused permanent hoarseness, in consequence of which I was unable to preach. I had no cough—no pulmonary disease. The whole difficulty seemed to be located in the larynx. I am not aware that any medical treatment was of any service, and I soon relinquished all attempts to remove the difficulty by medicine. The hoarseness would come on suddenly, as though by a cold, and leave as suddenly, of its own accord, and usually as soon without, as with medicine. My general health was tolerably good; and, as I had previously studied medicine two years, I completed my medical education and commenced practice in Boston, where I practiced nearly twenty years. A part of this time my voice was clear and strong; but I could place no reliance upon it, as I would be able to speak in public one day, and so hoarse the next that I could scarcely be understood.

In 1852 I spent a winter in Philadelphia. While there I had much less hoarseness than I usually had in Boston. In 1859 I

removed from Boston to Philadelphia, chiefly on account of this difficulty, and I remained there several years. During that time I had very little hoarseness. I then returned to Boston. Last winter I had two severe attacks of my old complaint, from the last of which I did not recover till I went to Philadelphia, and in two weeks I was free from the complaint and my voice good. I have been more or less hoarse several times the past summer in Boston, and at this present time, September, 1867, am laboring under a severe attack of my old malady. What there is in the Boston atmosphere to cause the difficulty, or in the air of Philadelphia to remove it, I do not know. I know only the facts.

I have thus gone into my personal history to prepare the way to state what I have found the most benefit from in the way of atomized medical inhalation. Perhaps I ought to add, during these thirty years I have not been sick or laid aside a day, nor gained nor lost two pounds of flesh, so well balanced have been the loss and the repair.

Your readers must not infer from this statement that Philadelphia is generally a more healthy city than Boston; for, while it is a better climate for lung and throat diseases, as it proved in my own case, it is not so favorable to disease operating upon the apparatus of digestion; and, while I was free from any trouble of the air passages, my wife lost her health and became very feeble by a nervous dyspepsia. But she has improved since our return to Boston.

I had found some benefit from the powder named above, in my own case. I had also employed alum in the same way; but in my case it was not as beneficial as the argentum. When the *atomizer* was first invented I tried the alum and the argentum in my own case, but derived only small benefit from their use—not half so much in my own case as I have seen in many others. I have received more benefit from muriate of ammonia, grains x to aqua ℥i, and inhaled three or four times daily, than from any or all other remedies. I had been vexed with a catarrh, hoarseness, and more cough than I ever had before. It had been growing no better for ten days, during which time I had inhaled atomized alum, argentum, and various narcotic medicines. I tried a very weak solution of muriate of ammonia, only grains v to ℥i of water.

The effect was very perceptible. The voice immediately began to improve; the cough loosened, expectoration was facilitated, the head felt better, and the general symptoms were all mitigated. I then increased the dose to grains x to aqua ℥i, and found still more improvement, so that I have since tried no other remedy.

I had purposed to write out from my note book a number of cases, with the various results of treatment; but perhaps I have written enough for one number of your Journal. I will send the others for a future one, if you think it best to publish this statement.

